# Mineral-Forming Effect of the Joint Participation of Natural Infusible Calcium Silicate and Dust-like Silica in Ceramic Compositions

**DOI:** 10.3390/ma18132991

**Published:** 2025-06-24

**Authors:** Mukhtar Yendibayevich Kurbanbayev, Begen Omarovich Yessimov, Vladimir Ivanovich Vereshchagin, Tatyana Amanovna Adyrbayeva, Yelena Sergeevna Dubinina

**Affiliations:** 1 Department of “Silicate Technologies and Metallurgy”, Higher School “Chemical Engineering and Biotechnology”, M. Auezov South Kazakhstan Research University, Shymkent 190016, Kazakhstan; muk81981@mail.ru (M.Y.K.); tatianaadyrbaeva@mail.ru (T.A.A.); 2 Scientific and Educational Center N.M. Kizhnera, Engineering School of New Manufacturing Technologies, National Research Tomsk Polytechnic University, Tomsk 634050, Russia; vver@tpu.ru

**Keywords:** wollastonite, marshalite, burning, mullite, microstructure, electrical porcelain

## Abstract

Original compositions of electrical ceramics have been developed and tested using marshalite and wollastonite as raw materials. An analysis of the equilibrium states of the created porcelain masses at different temperatures in Na_2_O-Al_2_O_3_-SiO_2_ and K_2_O-Al_2_O_3_-SiO_2_ systems was carried out. The amount of melt in these systems was calculated based on equilibrium flux curves. The characteristics of the sintering process of the masses were identified. A scheme for the formation of key secondary needle-like mullite during the thermal treatment of the masses was outlined and the temperature intervals for the formation of intermediate compounds were found. X-ray diffraction patterns and micrographs of the synthesized samples were decoded, and the phase composition and microstructure of the samples were analyzed. The effective influence of silica component dispersion on the mineral formation processes during the sintering of the porcelain masses in model samples of feldspar compositions with quartz sand and marshalite was noted. The optimal firing temperatures for full mineral formation and structure formation have been determined, as well as the physical–mechanical and dielectric properties of the obtained ceramic samples.

## 1. Introduction

Electrical porcelain is a type of ceramic material that functions as an electrical insulating element. Electrical porcelain products are very diverse in their chemical composition, properties and purpose. As is known [1,2,3,4,5,6], there are three classic types of ceramic compositions of electrical insulating porcelain: feldspar ordinary, quartz and aluminous. Due to the diversity of insulator designs, their production requires different initial ceramic masses [1,2,3,4,5,6], different technologies [1,2,3,4], modes [7,8] and equipment. According to its purpose, electrical porcelain is divided into low-voltage and high-voltage, the latter of which is designed to operate at voltages above 1000 V [1,2,3,4]. The main electrical and physical-mechanical characteristics of high-voltage products—electrical strength, dielectric constant, dielectric loss tangent, porosity, flexural and compressive strength—are ensured by careful selection and determination of the correct ratios of the components of raw materials [1,9,10,11,12,13,14,15,16,17,18,19].

The main raw materials for the production of porcelain products are white-burning clays, kaolins, quartz and feldspar rocks [1,2,3,9,10,11,12,13,14,15,16,17,18,19]. Each component of the mass has a specific role in the ceramic mass. Kaolin is introduced into the composition of porcelain masses in raw and fired form [1,2,3,4,16,20]. The role of kaolin in porcelain mass consists mainly in increasing the mechanical, thermal and chemical stability of the product and in giving it whiteness. This role is manifested in connection with the dissolving action of feldspar. Due to the diffusion of aluminum ions from the dissolved dehydrated kaolin into the feldspar melt, their concentration in the melt increases and the main condition for the occurrence of the mullite phase in it is created. Both mullite and the melt hardened upon cooling, enriched with aluminous and siliceous components, improve the mechanical, thermal and chemical properties of porcelain [20,21]. The amount and purity of the introduced kaolin directly affect the whiteness of the products [1,20,21]. Clays impart plasticity to the masses and are sintered in such a way that the hardening and compaction that occurs during this process do not cause the products to lose their shape [1,2,3,4,10,12,14,22,23].

The role of the silica component is as follows. Quartz as a leaning material helps to reduce air and fire shrinkage of products [15,24]. Dissolution of quartz in feldspar glass, as well as kaolin, causes an increase in refractoriness, viscosity, and helps to increase the resistance of the product to deforming forces during the firing process [1,2,3,4,10,12,14,25]. During the sintering process, it interacts with other components of the fired porcelain mass to form new compounds that determine the technical properties of the resulting porcelain; quartz also imparts translucency to the products [1,12,25].

The feldspar component in the porcelain mass ensures the development of the glassy phase, which acts in three ways [12,14,16,19,26,27,28,29]. Firstly, it dissolves other components of the mass; secondly, it imparts pyroplasticity and strength to the material during firing and promotes the crystallization of mullite [1,2,3,4,12,14,16,19].

It is known [1,12,27,30] that the technical characteristics of porcelain products can be improved by finer grinding of the initial raw materials. With an increase in the dispersion of lean materials, the mechanical and electrical strength of porcelain increases significantly, the homogeneity of the material structure improves, chemical transformations are accelerated during solid-phase interactions and liquid sintering, the amount of quartz grains in the residue decreases, thereby increasing mechanical strength and heat resistance [30,31,32,33,34]. Improving the properties of porcelain can also be achieved by using mineral additives—so-called mineralizers [7,17,27,35,36,37,38], introduced in small quantities into the composition of the mass. They have a significant impact on the technical characteristics of finished products. Their presence in ceramic masses reduces the sintering temperature and significantly enhances mullite formation in porcelain, which directly affects the properties of the finished product [9,16,36].

The growing demands on the dielectric and physical-mechanical properties of electrical insulating porcelain products, associated with the steady increase in the production and consumption of electricity, leads to the need to search for new compositions of electrical porcelain masses [14,16,17,19].

A review of scientific literature has shown that numerous studies on obtaining porcelain with improved properties are aimed at developing mass compositions using porcelain waste as quartz-containing components [16,17,35], ashes of various materials [15], silica fume [31], marshalite [25,39] and various wastes [16,17,18,19,20], and diopside is used as a mineralizer in them [1,22,35,37,38,40,41], talc [10,12], calcite and other minerals containing oxides of calcium, magnesium and barium [37,38]. There are no studies on the combined effect of marshallite and wollastonite additives on the sintering processes, formation of the structure and properties of porcelain for electrical purposes [42].

This work is based on research into the use of natural highly dispersed quartz—marshalite—as the most effective silica component in electrical porcelain masses and the influence of even a small proportion of wollastonite doses on the formation of the structure and properties of ceramics.

## 2. Materials and Methods

### 2.1. Raw Materials

The objects of the study are selected domestic types of mineral raw materials, experimental ceramic masses based on kaolin Soyuzny deposit, white-burning clay of Berlin deposit, feldspar of Sarybulak deposit, quartz sand of Mugodzharsky deposit, marshalite of Mansurata deposit, wollastonite from the Vekhne-Badam deposits and created samples of electrical porcelain [42].

#### 2.1.1. Kaolins

Based on the analysis of the chemical and mineralogical composition and properties of Kazakhstan kaolins, kaolins from the Soyuzny deposit (Aktobe region, Kazakhstan) were selected for research [24,43].

The determination of the mineral composition of kaolins is based on a comprehensive determination of the qualitative mineral composition of clay raw materials with the help of X-ray (DRGP-3 diffractometer using data from the ICDD card index: powder diffractometric data base PDF 2 (Powder Diffraction File) ICDD-2, Release 2022, San Francisco, CA, USA), thermographic (D-102 derivatograph of the F. Paulik, I. Paulik, L. Erdey system, Hungary) analysis.

Chemical composition of enriched kaolins from the Soyuzny deposit, % by weight: SiO_2_—50.69, Al_2_O_3_—36.34, Fe_2_O_3_—0.0, TiO_2_—0.51, CaO—0.29, MgO—0.08, K_2_O + Na_2_O—0.17, loss on ignition—11.88 [24,43,44].

#### 2.1.2. Clays

Based on the analysis of the chemical and mineralogical composition and properties of Kazakhstan clays, clays of the Berlin deposit (Kostanay region, Kazakhstan) were selected for research [24,44].

The determination of the mineral composition of clays is based on a comprehensive determination of the qualitative mineral composition of clay raw materials with the help of X-ray (DRGP-3 diffractometer using data from the ICDD card index: powder diffractometric data base PDF 2 (Powder Diffraction File) (ICDD-2, Release 2022, San Francisco, CA, USA), thermographic (D-102 derivatograph of the F. Paulik, I. Paulik, L. Erdey system, Hungary) analysis [42].

Chemical composition of clays of the Berlin deposit, % by weight: SiO_2_—49.69, Al_2_O_3_—31.02, Fe_2_O_3_—0.85, TiO_2_—0.42, CaO—0.61, MgO—0.07, K_2_O—0.78, Na_2_O—0.11, loss on ignition—14.92 [24].

#### 2.1.3. Feldspars

In our work, feldspars from the Sarybulak deposit (Zhambyl region, Kazakhstan) were used as fluxes in the technology of production of electrical porcelain [27,43].

Determination of the qualitative mineral composition of feldspars was carried out using the X-ray analysis method (DRGP-3 diffractometer using data from the ICDD card index: powder diffractometric data base PDF 2 (Powder Diffraction File) (ICDD-2, Release 2022, San Francisco, CA, USA). The chemical composition of feldspars is characterized by the following composition, % by weight: SiO_2_—78.78, Al_2_O_3_—10.55, Fe_2_O_3_—0.47, CaO—0.14, K_2_O—6.02, Na_2_O—4.04 [42].

#### 2.1.4. Quartz Sands

Quartz sands from the Mugodzharsky deposit (Aktobe region, Kazakhstan) were considered as nonplastics materials [24,44].

Determination of the qualitative mineral composition of quartz sands was carried out using the X-ray analysis method (DRGP-3 diffractometer using data from the ICDD card index: powder diffractometric data base PDF 2 (Powder Diffraction File) (ICDD-2, Release 2022, San Francisco, CA, USA). Chemical composition of quartz sands of Mugodzharsky deposit contains, % by weight: SiO_2_—98.2, Al_2_O_3_—0.4, Fe_2_O_3_—0.11, TiO_2_—0.05, CaO—0.14, MgO—0.09, K_2_O—0.17, Na_2_O—0.17 [24].

#### 2.1.5. Marshalite

Marshalites are used as a substitute for quartz sand in porcelain masses; we studied the marshalites of the Mansurat deposit (Turkestan region, Kazakhstan) [44].

According to X-ray phase analysis, silica in marshalites is represented by quartz (89.7%), and kaolinite lines (10.3%) are also recorded on the X-ray diffraction pattern. Chemical composition of marshalites of the Mansurata deposit, % by weight: SiO_2_—95.8, Al_2_O_3_—4.06, Fe_2_O_3_—0.14 [43]. According to physical characteristics, the marshalites of the Mansurata occurrence are a free-flowing powder, which is characterized by a high content of SiO_2_. The main difference from quartz sands is a relatively high specific surface area and low density. Marshalites are characterized by homogeneity and consist mainly of angular unrolled quartz grains. The content of particles larger than 0.01 mm is about 10–15%. The low content of ferrous inclusions, as well as the presence of kaolinite, which has a beneficial effect on the formation of the microstructure of the shard, indicate the possibility of using Mansurata marshalites in the technology of electrical porcelain without enrichment [42].

#### 2.1.6. Wollastonite

The Verkhne-Badam wollastonite deposit is located in the Turkestan region of Kazakhstan [44]. The samples we examined contain wollastonite (97.8%), calcite (1.3%) and quartz (0.9%). As the analysis of the chemical and mineralogical composition and technological properties shows, wollastonite from the Verkhne-Badam deposit has high quality indicators and is of interest for use as additives in porcelain masses [44].

In determining the composition and properties of wollastonites, methods for determining the chemical composition and physical properties of feldspar and quartz-feldspar materials were used in accordance with standards.

### 2.2. Preparation of Ceramic Masses, Molding of Samples

The preparation of ceramic masses was carried out in accordance with classical methods [1,6,45].

In laboratory conditions, a plastic molding method (humidity 20–22%) was chosen for the production of experimental samples of electrical insulating products.

The technological characteristics of ceramic masses are determined in accordance with the requirements of GOST 21216-2014 “Clay raw materials. Test methods”, generally accepted methods for studying ceramic masses [45,46].

The molded products were dried to a moisture content of 1–2%.

### 2.3. Heat Treatment of Semi-Finished Products

Firing of the experimental samples was carried out in a high-temperature laboratory furnace (NABERTHERM HTC, HTCT 08/16, Lilienthal, Germany).

The studies of the sintering properties of the developed ceramic masses were carried out in accordance with the requirements of GOST 21216-2014 “Clay raw materials. Test methods” [45,46], generally accepted methods for studying ceramic masses.

We have adopted the following firing mode for a pilot batch of electrical products. Firing is divided into several periods:

- the first period occurs with a temperature rise to 800–830 °C. During this period, very little sintering of the material is achieved. Within the range from 20 °C to 800 °C, the temperature rises at a rate of 150–200 °C per hour with a holding time of 550–600 °C;
- the second period begins with 800–850 °C and ends with a temperature of 1000–1080 °C. It is characterized by a slow rise in temperature of 30–50 °C per hour;
- in the third period, the temperature rises to 1350 °C at a rate of 60–100 °C per hour and ends with a 2–3 h hold at the final temperature (1350 °C);
- the fourth period is cooling, the temperature is reduced to 800–700 °C at a high speed (200–250 °C/hour), and then the cooling rate decreases.

### 2.4. Determination of the Main Technical Indicators of Synthesized Samples of Electrical Porcelain, Studies of the Chemical and Mineralogical Composition and Microstructure

The main technical parameters of the synthesized samples of electrical porcelain are determined in accordance with the requirements of standards [47].

To determine the mineral composition of the synthesized samples, the X-ray analysis method was used. X-ray analysis was carried out on an automated DRON-3 diffractometer with CuKa radiation, β-filter. Conditions for recording diffraction patterns: U = 35 kV; I = 20 mA; shooting θ–2θ; detector 2 deg/min. X-ray phase analysis on a semi-quantitative basis was performed on the diffraction patterns of powder samples using the method of equal weighed portions and artificial mixtures. Quantitative ratios of crystalline phases were determined. The interpretation of diffraction patterns was carried out using data from the ICDD card index: powder diffractometric data base PDF 2 (Powder Diffraction File) (ICDD-2, Release 2022, San Francisco, CA, USA) [42].

The studies of the chemical and mineralogical composition and microstructure of the synthesized samples were carried out using scanning electron microscopy. The work used a modern multi-purpose scanning electron microscope of the JSM-6490LV series (JEOL, Ltd., Tokyo, Japan, 2007) with powerful software, computer control. The JSM-6490LV electron microscope has 2 attachments (Oxford Instruments, Abingdon, UK) for energy-dispersive microanalysis INCAEnergy350, texture analysis of polycrystalline and crystalline samples HKL Basic [42].

## 3. Results and Discussion

In this work, we show the mineral-forming effects of the joint participation of two mineral types of raw materials with unusual physical and chemical properties that have rarely been studied in regard to this technology—wollastonite of contact-metasomatic origin and quartz of a finely ground to powdery state, caused by natural exogenous processes. The emerging features of the formation and transformation of the phases, structure, and physical and technical properties of the synthesized electrical porcelain were investigated.

A series of laboratory studies were carried out in the course of searching for more rational compositions of masses. New compositions were selected based on calculations and experiments. The standard composition 1SC with the following components was adopted as the starting point: kaolin (27.5%), white-burning clay (22.5%), quartz sand (18.0%) and feldspar (32.0%) [42].

In order to establish and analyze the dynamics of changes in the characteristics of this ceramic in the process of introducing selected components for experiments, three potentially interesting compositions were found based on the preliminary results:2CM, 3CW and 4CMW. In the 2 CM composition, traditional quartz sand (18%) is completely replaced by marshalite; in 3CW wollastonite (2%) is used; and in 4CMW, where quartz sand (18%) is also completely replaced by marshalite, wollastonite is also introduced (2%).

There are scientific papers [31,48] in favor of using finely dispersed silica, such as «Iraqi porcelanite» and silica fume, as a replacement for traditional quartz, which is of environmental interest and allows the production of ceramic products with better mechanical properties. Comprehensive studies of ceramic masses have shown [49] a positive effect of using quartz and feldspar in the form of fine powders with a particle size of 1.2 microns, expressed in a decrease in the sintering temperature and a significant increase in the bending strength of products, and a more striking effect of quartz in this regard has also been noted.

The introduction of mineralizing additives into ceramic masses in small quantities leads to an improvement in the porcelain formation process and an increase in the appearance of mullite crystals. The latter, in turn, improve the quality characteristics of finished products [5,25,50,51,52,53,54].

It is known [1,25] that the conductivity of porcelain insulators has a mixed ion-electronic character. The ionic component of conductivity is determined by alkaline ions Na^+^ and K^+^, and alkaline earth ions are practically not involved in conductivity up to 1000 °C. In a direct current field, the phenomenon of electrolysis occurs in porcelain. And its products Na^+^ and K^+^ are oxidized and can enter into secondary reactions with the material, which usually causes breakdown of insulators. Research by some authors [40,53,54,55,56] has confirmed the feasibility of replacing alkaline ions in porcelain with alkaline earth ions, in particular, Ca^2+^ ions. This has a beneficial effect on the electrical and physical-mechanical properties of electrical porcelain.

To obtain a preliminary assessment of the sinterability of the masses, melting curves were constructed using the phase diagrams of the K_2_O-Al_2_O_3_-SiO_2_and Na_2_O-Al_2_O_3_-SiO_2_ systems. The choice of two systems is explained by the insignificant difference in the K_2_O and Na_2_O contents in the masses.

The positive value of the phase diagram is that it makes it possible to determine both the sequence of separation of solid phases and the limiting state to which the system tends. The analysis of the equilibrium states of porcelain masses at different temperatures in the Na_2_O-Al_2_O_3_-SiO_2_ and K_2_O-Al_2_O_3_-SiO_2_ systems is due to the fact that the contents of other oxides do not exceed 0.5% and in total amount to 1.38%. The developed compositions under consideration are located within the SiO_2_–3Al_2_O_3_∙2SiO_2_–K_2_O∙Al_2_O_3_∙6SiO_2_ triangle.

**Figure 1:**
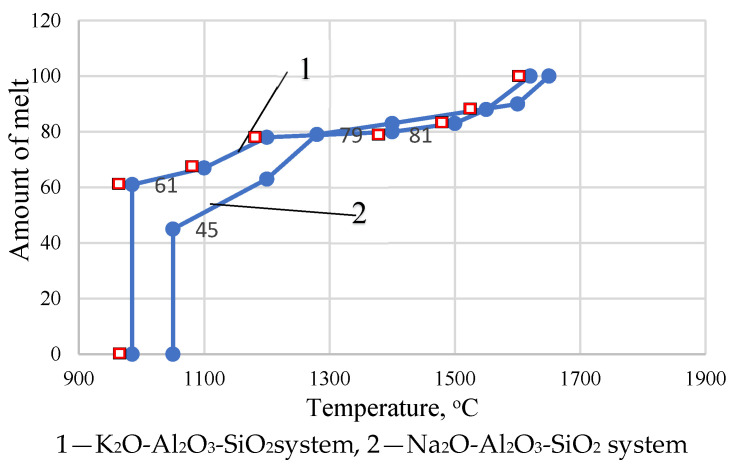
Equilibrium melting curves of the studied masses.

The amount of primary melt formed in the masses in the Na_2_O-Al_2_O_3_-SiO_2_ system is 45% (1050 °C), and in the K_2_O-Al_2_O_3_-SiO_2_ system is 61% (985 °C). According to the equilibrium melting curves of the above systems, at a firing temperature of 1270–1350 °C, 79–81% of the melt is formed (In each case, several samples were tested. The standard deviation error is 0.9%) (Figure 1).

The actual amount of melt during the firing of the studied masses does not correspond to the amount of melt calculated from the equilibrium melting curves. Ternary eutectics are not formed in systems at temperatures of 985–1050 °C. This is due to the fact that mullite is not synthesized below a temperature of 1200 °C.

Insignificant shrinkage (5–6%) of porcelain masses up to a temperature of 1000 °C is associated with solid-phase sintering and the appearance of a primary melt in small quantities due to binary eutectics. The equilibrium amount of eutectic melt in the K_2_O-SiO_2_ system at a temperature of 767 °C is 6.55%, and the amount of eutectic melt in the equilibrium state in the Na_2_O-SiO_2_ system at a temperature of 793 °C is 4.07% [39,42]. The total amount of melt in the equilibrium state at a temperature of 793 °C is 10.62% (Table 1).

**Figure 2:**
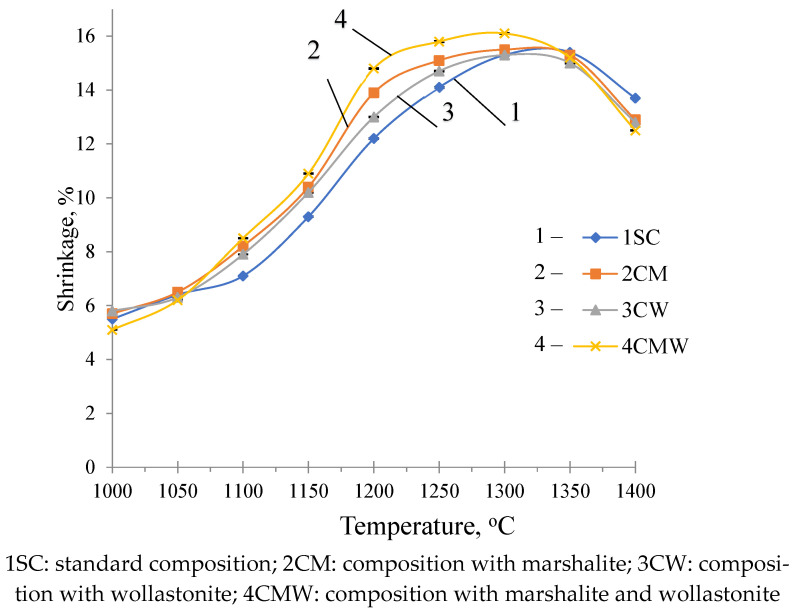
Shrinkage of test samples fired at different temperatures.

On the shrinkage curves, intensive sintering is recorded from a temperature of 1070 °C, shrinkage at this temperature increases from 5–6% to maximum values (14–16%) at firing temperatures of 1250–1300 °C (In each case, several samples were tested. The standard deviation error is 0.18%) (Figure 2).

When the studied mass is fired in the solid phase, the decomposition of clay minerals occurs, mainly kaolinite, which makes up 42.5%. Its decomposition occurs at 570–580 °C, and at a temperature of 975–980 °C, a spinel phase is formed according to the following reaction scheme [39,42]:

$$\begin{array}{l} \underset{\mathrm{kaolinite}}{{\mathrm{Al}}_{2}O_{3}\cdot 2{\mathrm{SiO}}_{2}\cdot 2H_{2}O}\overset{570–580\;{}^{\circ}C}{\to }\underset{\mathrm{metakaolinite}}{{\mathrm{Al}}_{2}O_{3}\cdot 2{\mathrm{SiO}}_{2}+2H_{2}O}\overset{975–980\;{}^{\circ}C}{\to }\underset{\mathrm{silicon}\;\mathrm{spinel}}{{2\mathrm{Al}}_{2}O_{3}\cdot 3{\mathrm{SiO}}_{2}+{\mathrm{SiO}}_{2}}\overset{>1100\;{}^{\circ}C}{\to }\\ \to \underset{\mathrm{pseudo}\;\mathrm{mullite}}{2({\mathrm{Al}}_{2}O_{3}\cdot {\mathrm{SiO}}_{2})+{\mathrm{SiO}}_{2}}\overset{>1200\;{}^{\circ}C}{\to }\underset{\mathrm{mullite}}{3{\mathrm{Al}}_{2}O_{3}\cdot 2{\mathrm{SiO}}_{2}}+{\mathrm{SiO}}_{2}+melt \end{array}$$

The oxide system in this case does not reach equilibrium due to the fact that the mullitization process takes place at temperatures above 1200 °C. Interactions with components and the dissolution of quartz is expanded in time and temperature. For the activation of phase formation with the participation of silica, the quartz sand in the masses was completely replaced by marshalite. Marshalite is a natural highly dispersed silica raw material, where the proportion of particles smaller than 0.01 mm is 80–85%, while the average particle size of quartz sand in the mass, even after grinding, is 25–30 microns [42].

**Figure 3:**
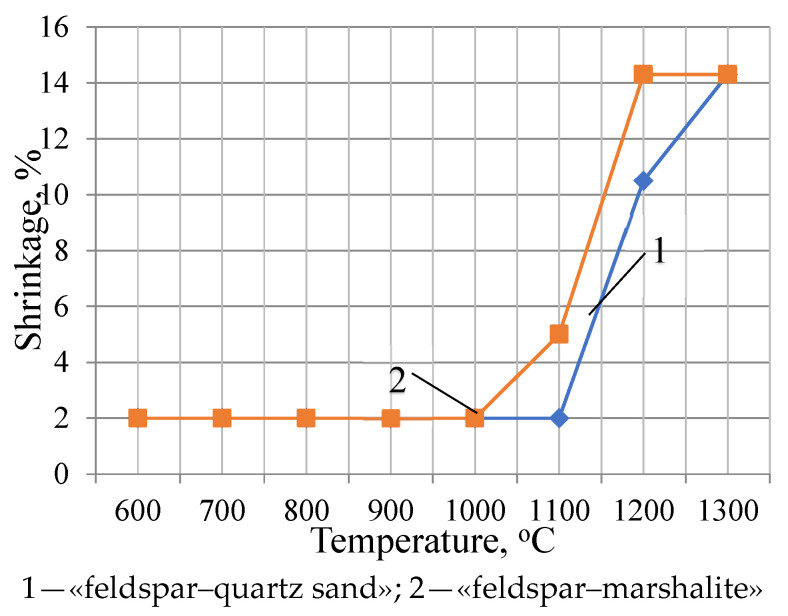
Shrinkage of experimental samples of compositions fired at different temperatures.

The influence of the dispersion factor on the sintering processes, accompanied by active mineral formation, is demonstrated in the shrinkage and water absorption curves of the «feldspar–quartz sand» and «feldspar–marshalite» compositions we studied [8,9,12]. The preparation of the investigation samples was carried out using the same technology as the studied porcelain masses. The burning of these samples was carried out at temperatures of 600–1300 °C every 100 °C. The shrinkage curves of the samples of the studied compositions are presented in Figure 3. In the temperature range of 600–1000 °C, changes in the linear dimensions are insignificant, which is characterized by the straight line on the graph corresponding to 2% shrinkage. An increase in the shrinkage value for the compositions with marshalite is observed starting from a firing temperature of 1000 °C. The maximum value (14.3%) of shrinkage is reached at a firing temperature of 1200 °C, and this value remains unchanged at a temperature of 1300 °C (In each case, several samples were tested. The standard deviation error is 0.15%) [39,42,47].

The shrinkage of the samples of the with quartz sand compositions up to a temperature of 1100 °C does not exceed 2%, a further increase in temperature is accompanied by an increase in shrinkage values up to 14% at a temperature of 1300 °C. The analysis of the shrinkage curves of the studied compositions showed that sintering of the compositions with marshalite begins at a temperature 1100 °C, which is almost 100 °C lower than the sintering temperature of the quartz–feldspar composition [39,42,49].

It becomes possible to determin of water absorption of the samples of the studied compositions starting from a firing temperature of 1000 °C, at which point they begin to acquire monolithicity.

**Figure 4:**
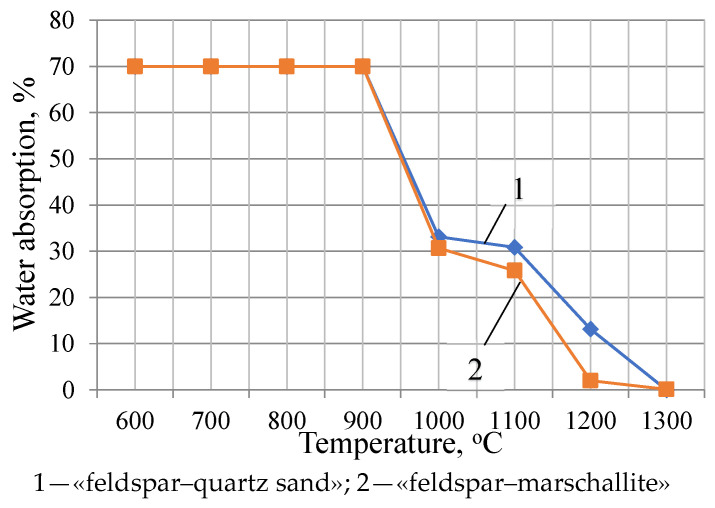
Water absorption of experimental samples of compositions fired at different temperatures.

The analysis of the dynamics of the water absorption of the samples of the studied compositions showed that the values of this indicator for the feldspar–quartz sand composition decrease uniformly, starting from a temperature of 1100 °C, then reach zero at a temperature of 1300 °C (In each case, several samples were tested. The standard deviation error is 0.05%) (Figure 4). The water absorption of the feldspar–marshalite composition approaches zero (2.0%) at a temperature of 1200°C, while that of the feldspar-quartz sand composition at this temperature is equal to 13.2%. The obtained results show that the finely dispersed marshalite actively interacts with feldspar, starting already from as low as 1000 °C [39,42,49].

**Figure 5:**
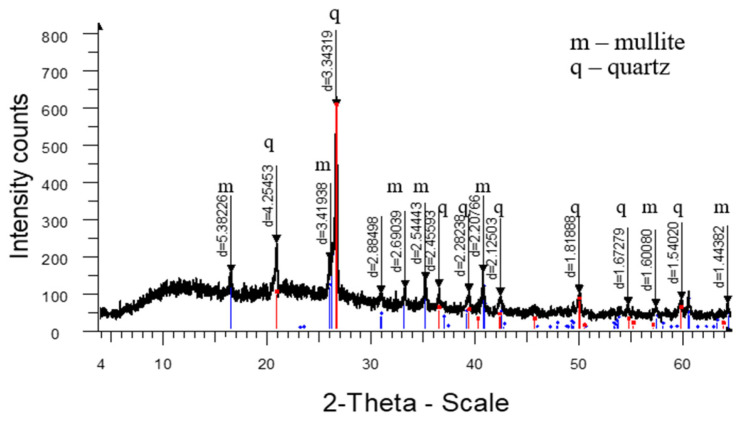
X-ray diffraction pattern of ceramics fired at 1320 °C from standard composition (1SC).

**Figure 6:**
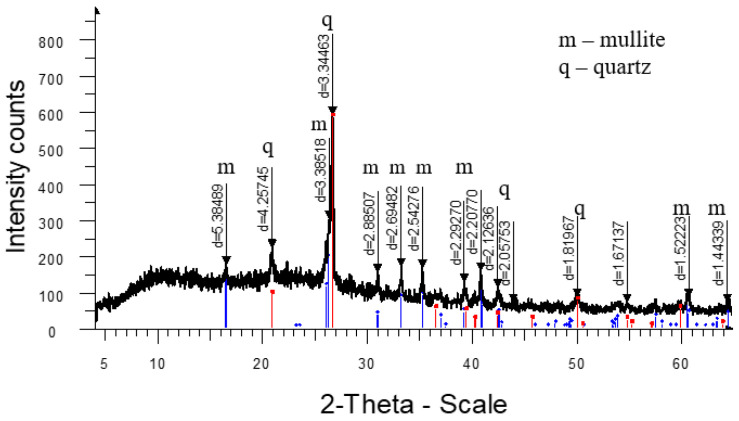
X-ray diffraction pattern of ceramics fired at 1290 °C from a mass with marshalite (2CM).

**Figure 7:**
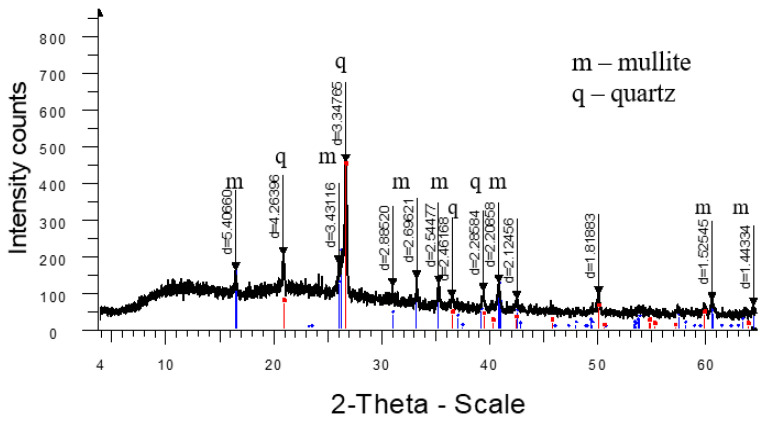
X-ray diffraction pattern of ceramics fired at 1300 °C from a mass with wollastonite (3CW).

**Figure 8:**
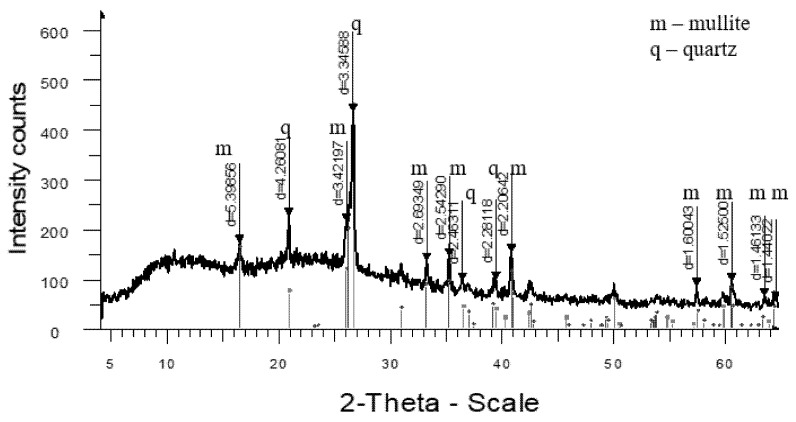
X-ray diffraction pattern of ceramics fired at 1270 °C from a mass with marshalite and wollastonite (4CMW).

The analysis of the high-temperature phase transformations in the developed mass compositions according to the X-ray diffraction patterns (Figure 5, Figure 6, Figure 7 and Figure 8) shows that the diffraction maximum of quartz (d = 4.2574; d = 1.8188) of the mass with marshalite (2CM) is 20–25% less than the similar indicator of the mass with quartz sand (1SC). Replacing quartz sand with marshalite leads to an intensification of the process of interaction of quartz with feldspar during the formation of eutectic melts up to a temperature of 1060 °C (binary eutectic) and the processes of dissolution of quartz in the melt at higher temperatures. An increase in the content of the glass phase in the mass with marshalite is also confirmed by the presence of diffraction scattering in the corresponding X-ray diffraction pattern (Figure 6). The appearance of the largest amount of glass phase is recorded in the mass with marshalite and wollastonite (Figure 8). The use of marshalite as a quartz-containing component and wollastonite additives (4CMW) in the studied masses contributes to an increase in mullite formation, which is confirmed by an increase in the intensity of mullite reflexes by 1.3 times compared to the masses with quartz sand (1SC).

The increase of the glass phase content in the mass with marshalite is also confirmed by the presence of diffraction scattering in the corresponding X-ray patterns. The appearance of the largest amount of glass phase is recorded in the mass with marshalite and wollastonite.

**Figure 9:**
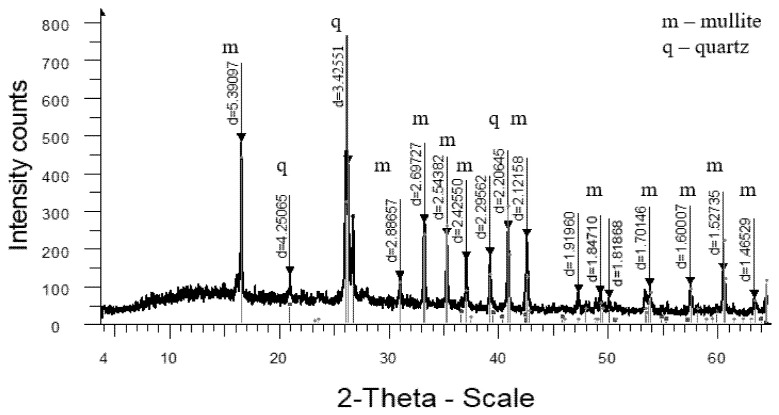
X-ray diffraction pattern of synthetic mullite.

For clarity in the comparative assessment, Figure 9 shows an X-ray pattern of reference synthetic mullite. The X-ray diffraction pattern shows slight diffraction scattering, indicating the negligible presence of glass phase. According to the data from the factory laboratory, in this sample the proportion of mullite in the crystalline phase reaches more than 92%, and for quartz more than 7%.

**Figure 10:**
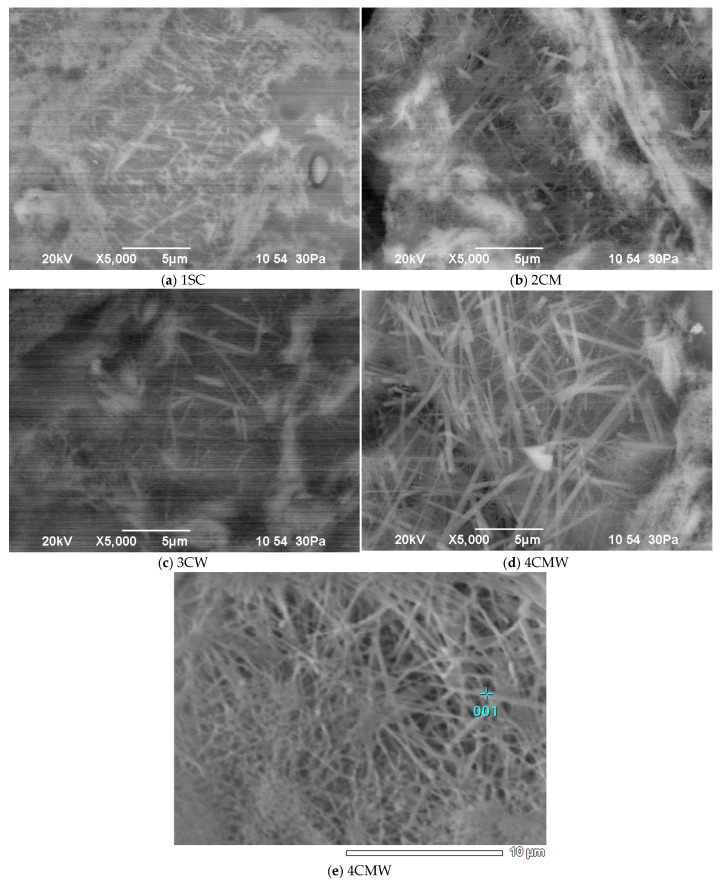
Micrographs of ceramic samples burned at the optimum temperatures. (**a**) 1SC—standard composition without additives; (**b**) 2CM—composition with marshalite; (**c**) 3CW—composition with wollastonite; (**d**) 4CMW—composition with marshalite and wollastonite and (**e**) 4CMW—composition with marshalite and wollastonite.

Using the capabilities of scanning electron microscopy, it was possible to obtain additional results on the phase composition, the nature of the crystalline neoplasms and the structure of the synthesized materials based on the compositions 1SC, 2CM, 3CW and 4CMW, subjected to firing at the found optimal temperatures (Figure 10).

Figure 10a shows one of the electron microscopic images of a chip of porcelain from a standard mass (1SC). The sample has a fairly dense structure, and its general appearance is represented by clearly distinguishable relics of feldspar from glass and mullite, grains of residual quartz, surrounded by halos of high-silica glass. Traces of the crystallization process of the feldspar-silica melt are clearly visible, which allowed for the growth of mullite aggregates, a mineral that is not typical in the earth’s crust for genetic reasons [39,42,49].

There are areas penetrated by fairly large well-developed columnar crystals and areas in which there is dense interlacing of clearly distinguishable mullite needles. The sizes of these crystals of new formations of aluminum silicate are mostly 2–3 microns (Figure 10a).

The images of the 2CM sample chip (Figure 10b) show a noticeable decrease in the size and amount of residual quartz, which confirms the increased reactivity of finely dispersed marshalite. Wares obtained using the 2CM composition have a more uniform and dense structure. Their main and constant part, in which the crystalline phases are dispersed, is a transparent base, i.e., the structurally glassy phase of the 2CM composition is a system intergrown with submicroscopic and uniformly dispersed mullite crystals [42].

The microstructure of the samples obtained on the basis 3CW composition (Figure 10c) is a complex heterogeneous system consisting of crystalline, glassy and gas phases. Glassy–mullitic regions are distinguished within the original feldspar particles and almost undecomposed particles of clay minerals. The noted mullite formations due to feldspar are completely different in size and growth pattern. Large mullite needles grow from the surface inward as the composition changes due to alkali diffusion in feldspar relics. The presence of quartz grains with a surrounding shell structure of glass mass is characteristic of this materials. The quartz surface is usually corroded by the feldspar melt. Mullitization extends right up to the outer edge of the glass phase zone.

The electron microscopic study of the samples of the 4CMW composition samples (Figure 10d,e) showed that the structure of the developed samples containing marshalite and wollastonite is characterized by a denser structure and the highest degree of mullitization. There are practically no pores in these products. The number and overall dimensions of the needle-shaped mullite crystals in the products, compared to those in other compositions, are noticeably increased due to the complex initiating effect of marshalite and wollastonite on the course of mineral formation [39,40,41,42,55,56,57].

Experiments show that in this ceramic material, replacing traditional quartz sand with marshalite helps to reduce the temperature of the liquid phase appearance, and wollastonite has a positive effect on the structure and properties of the binary eutectic melt. At the same time, diffusion processes involving the melt are activated, and its dissolving capacity increases. As a result, the amount of dissolved primary mullite increases and it is transformed into a crystalline phase in the form of secondary mullite, which is mostly represented in feldspar areas in the form of shagreen and thick felted fabric. Acicular secondary mullite is represented by crystals that are more perfect in shape and length, their length reaches 7–10 µm (Figure 10c,d), there are areas with densely intertwined and intergrow mullite needles that form a three-dimensional framework (Figure 10d,e). All of this ensures an increase in thermal resistance, physical, mechanical and electrical strength of the porcelain. Porcelain samples of composition 4SMW have a good degree of maturity [42].

**Figure 11:**
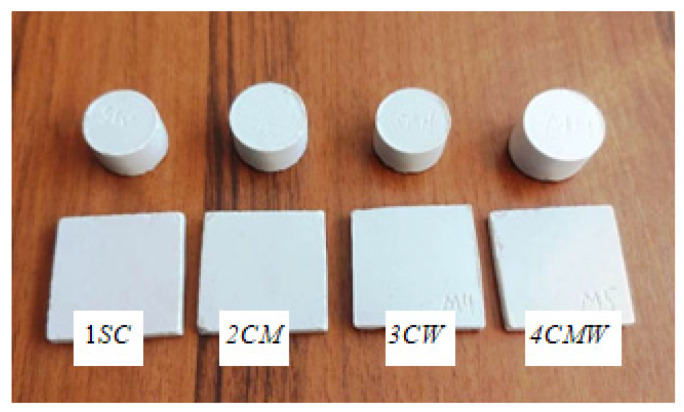
Ceramic samples synthesized based on the developed compositions.

The experimental ceramic samples obtained on the basis of the developed mass compositions, presented in Figure 11, have good physical, mechanical and electrical properties (Table 2).

The achieved transformations in the composition and structure of the developed ceramic materials had a positive effect on all of the properties encompassed by the standards (Table 2).

It should be noted that the firing temperature of the developed compositions was reduced to a useful level, it is in the range of 1270–1300 °C, while for the standard sample, it is 1340 °C.

The water absorption, shrinkage and density values of all the developed samples are normal and stable.

The electrical strength of products made from composition 1SC meets the requirements for ceramic electrical materials of group 100, and the highest indicator in this regard is for 4SMW composition. This is the result of the complex effect of marshalite and wollastonite. Replacing quartz sand in the mass with marshalite, which easily forms eutectics with orthoclase and albite, helps to reduce the melt appearance temperature. Inserting Ca^2+^ ions (1.04 Å) into the ceramic masses from the wollastonite structure activates the crystallization ability of mullite from this eutectic melt and replaces Na^+^ (0.98 Å) and K^+^ (1.33 Å) ions. As a result, the electrical strength increases. An increase in the number and length of the mullite crystals due to the action of wollastonite also has a positive effect on the mechanical strength of porcelain. The bending strength of the samples made of masses with marshalite and wollastonite is the highest and amounts to 81.7 MPa [39,42,58].

The thermal stability of the samples of the studied porcelain masses was determined by performing successive cycles of—heating in an electric furnace with a 30-min soak at a given temperature and abruptly cooling in water for 15 min. The results of determining the thermal stability of the samples showed the full compliance of their values with the requirements of State Standards. The highest thermal stability indicators were found in the 2CM and 4CMW compositions samples.

Thus, the introduction of original natural wollastonite and marshalite into ceramic masses, justified in terms of crystal chemistry, highly effectively affects the processes of mineral formation and the structure of ceramic stone structure formation.

## 4. Conclusions

It has been established that the sintering process of the developed ceramic compositions does not correspond to the equilibrium state. Ternary eutectics in the Na_2_O-Al_2_O_3_-SiO_2_ (1062 °C) and K_2_O-Al_2_O_3_-SiO_2_ (985 °C) systems are not formed. The discrepancy between the equilibrium melting curves and the actual sintering of the masses is the driving force of the sintering process of the studied masses.

Replacing quartz sand in the porcelain mass with highly dispersed marshalite reduces the porcelain sintering temperature by 50 °C to 1290 ± 10 °C due to the formation of eutectic melts in the K_2_O-SiO_2_ (767 °C), Na_2_O-SiO_2_ (793 °C) systems and eutectic melts of the quartz–orthoclase (990 °C) and quartz–albite (1062 °C) systems. The temperature of interaction of marshalite with feldspar in the developed masses approaches equilibrium and corresponds to temperatures of 1000–1050 °C [39,42,57,58].

The introduction of wollastonite (2 ± 0.2%) into the developed mass promotes the crystallization of needlelike mullite from the melt by reducing the viscosity of the melt. At the same time, the bending strength of the porcelain samples increases by 20% and reaches 75.9 MPa. The complete replacement of quartz sand in the composition of the masses with highly dispersed marshalite and the introduction of a small amount of wollastonite (2%) reduces the sintering temperature of the products by 70–80 °C to 1270 ± 10 °C compared to 1340 ± 10 °C for the standard 1SC mass. At the same time, the thermal strength increases by 22% (202 K), the bending strength increases by 29.1% (81.7 MPa) and the electrical strength increases by 29.0% (34.2 kV/mm) [39,42,57].

The formation of a eutectic melt at temperatures approaching equilibrium and an increase in the range of the sintered state due to finely dispersed silica marshalite, as well as a decrease in the viscosity of the melt due to Ca^2+^ ions of wollastonite create effective conditions for the crystallization of acicular mullite formations, which are so key for the formation of the material composition and structure of technical ceramics.

## Figures and Tables

**Table 1:** Compositions of low-temperature eutectics in binary systems and the equilibrium amount of melt.

System	Composition, Mass. %				Temperature, °C	Amount of Melt, %	
	Na_2_O	K_2_O	Al_2_O_3_	SiO_2_		At the Eutectic Point	Sum in Mass
K_2_O-SiO_2_	-	26.4	-	73.6	767	6.55	6.55
Na_2_O-SiO_2_	26.1	-	-	73.9	793	4.07	10.62
Al_2_O_3_-SiO_2_	-	-	5.5	94.5	1585	-	-

**Table 2:** Properties of the obtained ceramic samples.

Properties	State Standard Requirements	Compositions			
		1SC	2CM	3CW	4CMW
Burning temperature, °C	-	1340 ± 10	1290 ± 10	1300 ± 10	1270 ± 10
Water absorption, %	0.0	0.0	0.0	0.0	0.0
Shrinkage, %	-	15.5	15.7	15.1	16.1
Density, g/cm^3^	2.3	2.39	2.48	2.45	2.53
Bending strength, MPa	60	63.3	72.8	75.9	81.7
Electrical strength, 50 Hz, kV/mm	25	26.5	28.2	31.8	34.2
Thermal resistance, K	160	165	191	173	202
Interval of sintered state, °C	-	30–40	70–80	30–40	70–80

## Data Availability

The original contributions presented in this study are included in the article material. Further inquiries can be directed to the corresponding author.
